# Supplementary material for: Controlling thermal reactivity with different colors of light
Source: Nat Commun. 2017 Nov 30;8:1869. doi: 10.1038/s41467-017-02022-0 (PMC5707371; doi:10.1038/s41467-017-02022-0)
Supplement: Supplementary file 1 — Supplementary Information [file 41467_2017_2022_MOESM1_ESM.pdf]

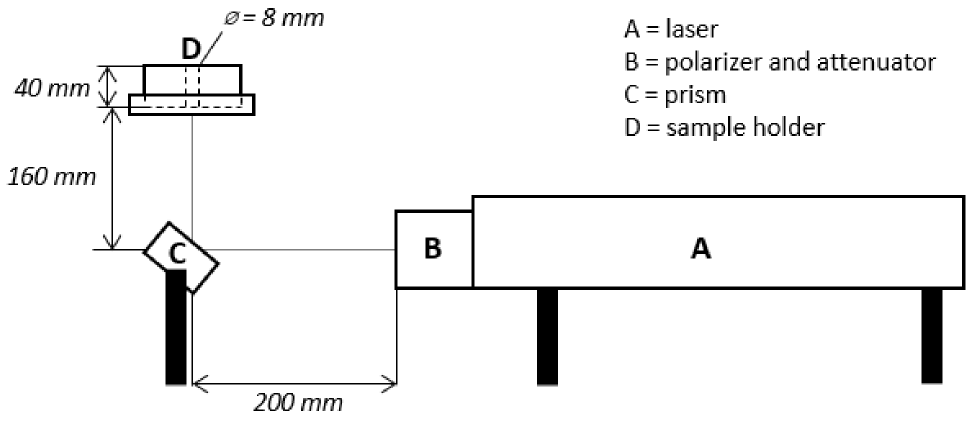

Supplementary Figure 1 | Schematic representation of the wavelength-tunable laser set-up used in this work for both UV- and visible light irradiation experiments. For detailed information, we refer to the instrumentation section.

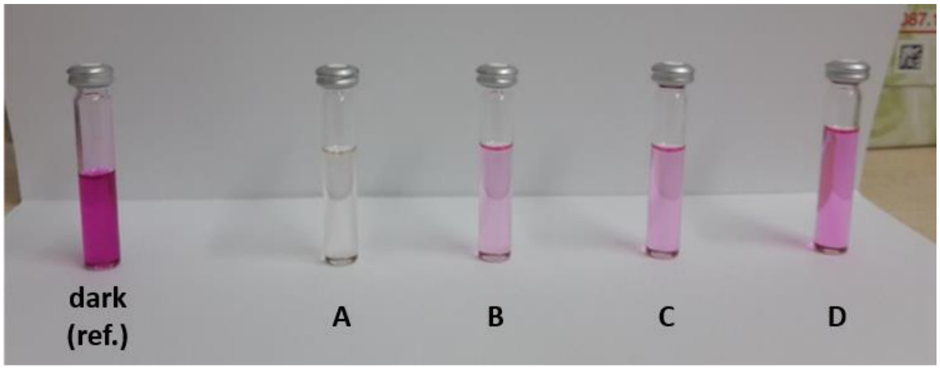

Supplementary Figure 2 | Irradiation of a 4-*n*-butyl-triazolinedione (**1**) solution in CCl_4 (0.3 M) for 10 minutes at 544 nm (4 mW cm^{-2}, 100 regenerated over time, here shown 15 (B), 30 (C) and 60 minutes (D) after irradiation.

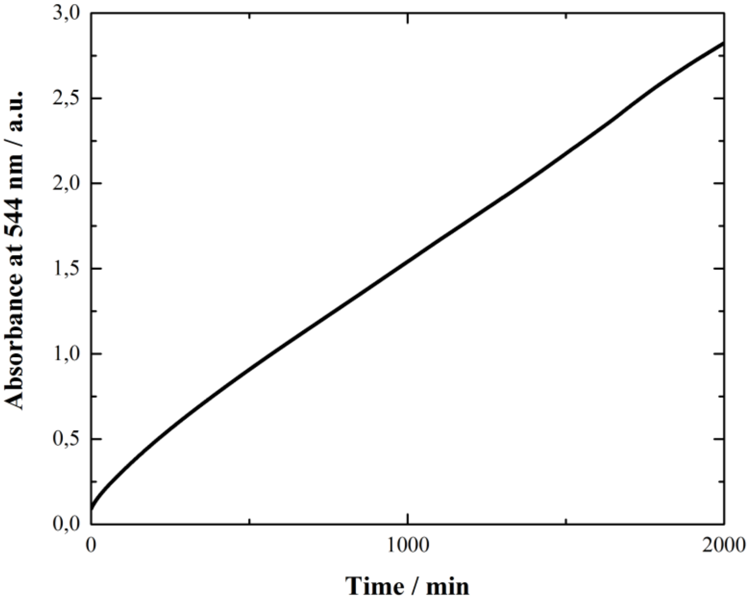

Supplementary Figure 3 | UV/vis measurement (CCl_4) after 10 minutes of visible light irradiation (544 nm, 4 mW cm^{-2}, 100 Hz) of a 0.3 M solution of **1** in CCl_4, qualitatively indicating the regeneration of the purple color over time.

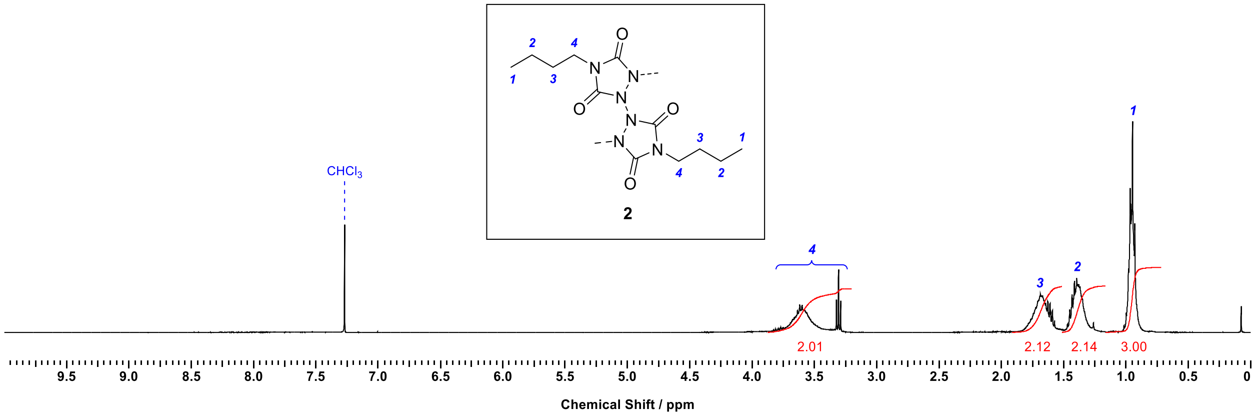

Supplementary Figure 4 | ^{1}H-NMR spectrum immediately after irradiation of **1** (0.3 M in $CDCl_3$) for 10 minutes at 544 nm (4 mW cm^{-2}, 100 Hz) shows characteristic peak broadening, indicating the formation of polymeric compound **2**.

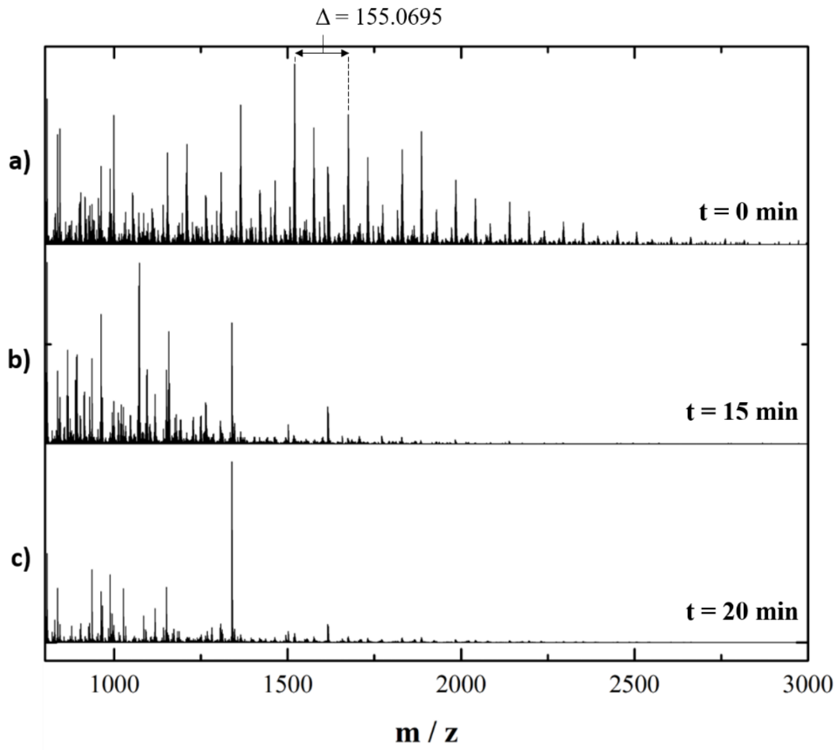

Supplementary Figure 5 | (a) ESI-MS spectrum recorded 2 minutes after irradiation of a solution **1** (0.3 M in $CDCl_3$) at 544 nm for 10 minutes (4 mW cm^{-2}, 100 Hz) indicating the presence of polymeric compound **2**. The fast disappearance of the polymeric signals after 15 min (b) and 20 min (c) upon standing in the dark demonstrate the thermal instability of the photopolymer.

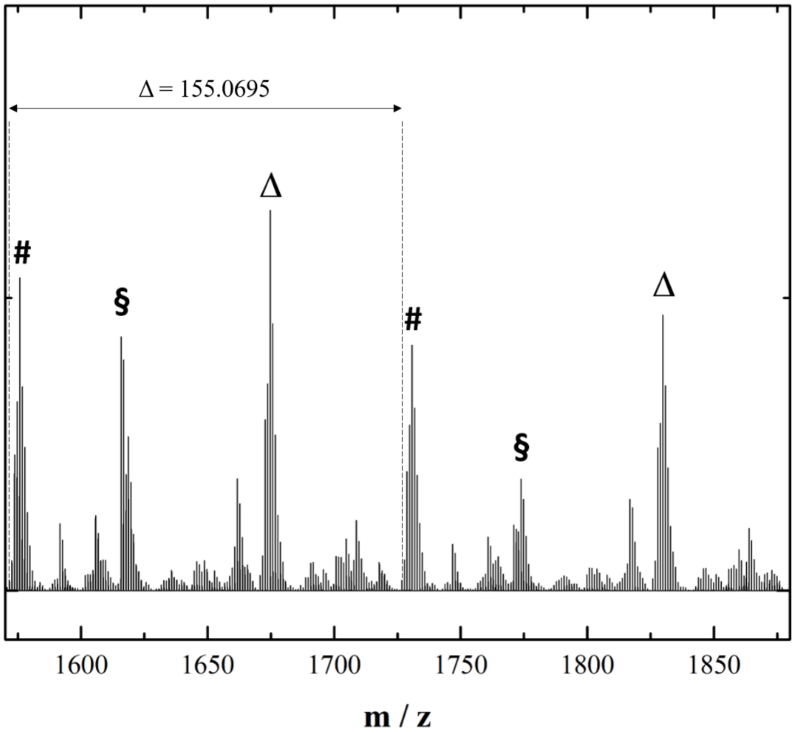

Supplementary Figure 6 | Zoom of the ESI-MS spectrum of photopolymer **2** formed after visible light irradiation.

Supplementary Table 1 | Theoretical and experimental m/z values (with a resolution R = 52 5000) observed in the ESI-MS spectrum of photopolymer **2**.

	# = $[2+Na]^+$			Δ = $[2^*+Na]^+$			§ = $[2^{**}+Na]^+$		
	$m/z_{theor.}$ (Da)	$m/z_{exp.}$ (Da)	Δ (Da)	$m/z_{theor.}$ (Da)	$m/z_{exp.}$ (Da)	Δ (Da)	$m/z_{theor.}$ (Da)	$m/z_{exp.}$ (Da)	Δ (Da)
**n = 9**	1418.6145	1418.6179	0.0034	1517.6830	1517.6864	0.0034	1616.7514	1616.7559	0.0045
**n = 10**	1573.6840	1573.6871	0.0031	1672.7524	1672.7685	0.0161	1771.8209	1771.8393	0.0184
**n = 11**	1728.7535	1728.7697	0.0162	1827.8219	1827.8395	0.0176	1926.8904	1926.9092	0.0188

Supplementary Table 2 | Plausible structures corresponding with the experimental m/z values found in the ESI-MS spectrum of photopolymer **2**.

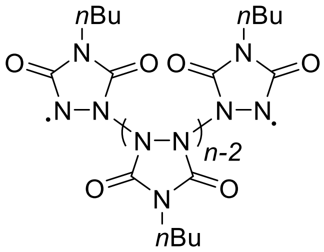

2

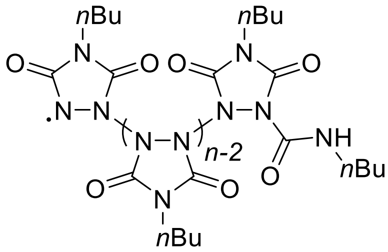

2*

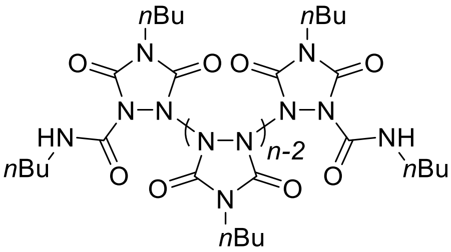

2**

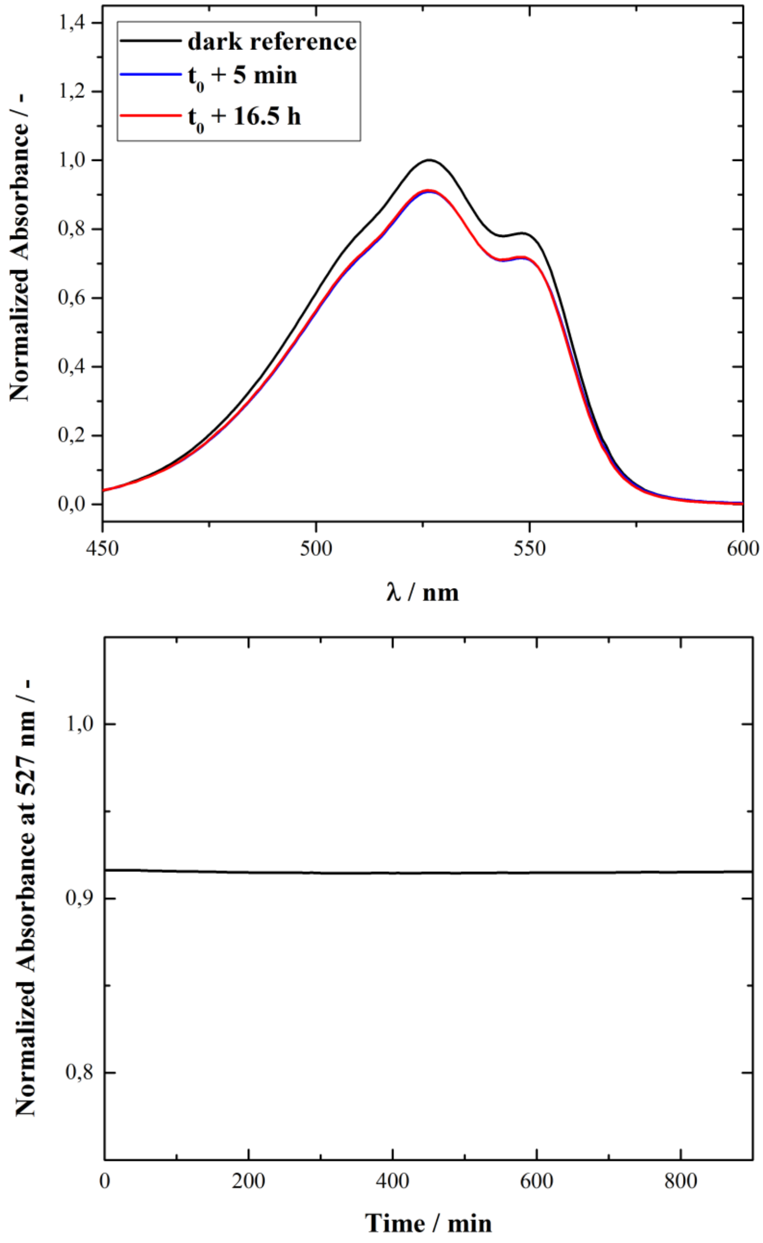

Supplementary Figure 7 | UV-vis spectrum (MeCN) after irradiation of **1** in acetonitrile (0.3 M, 1 h at 544 nm, 4 mW cm^{-2}, 100 Hz) only shows a minor decrease in intensity (which can be attributed to photodegradation) and thus no complete disappearance of **1**. Even after a prolonged time in the dark (16.5 h), no increase in absorbance (527 nm) and thus no depolymerization is observed.

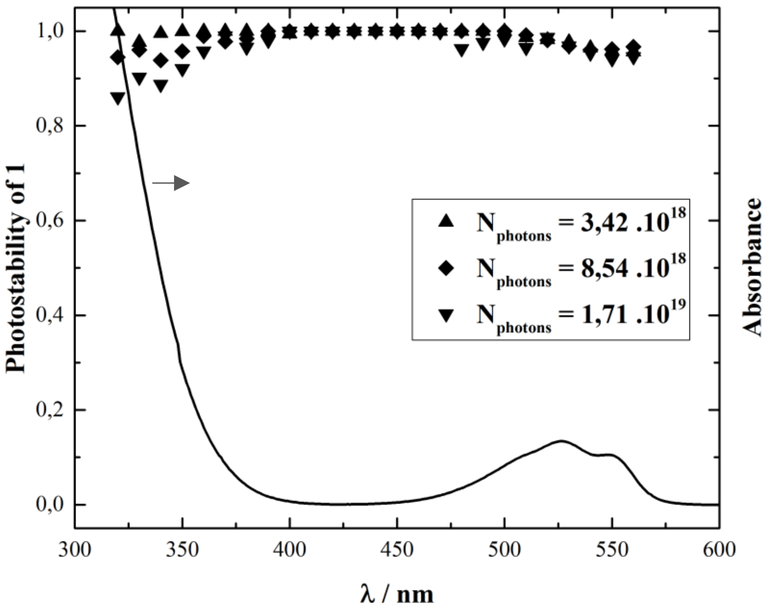

Supplementary Figure 8 | Photostability assay of **1** (MeCN, 5 mg mL^{-1}) in the wavelength regime of 320 to 560 nm with 10 nm intervals at a constant number of incident photons[1, 2] determined via UV/vis analysis (closed symbols, left y-axis) and overlay with the absorbance spectrum of **1** in acetonitrile (full line, right y-axis).

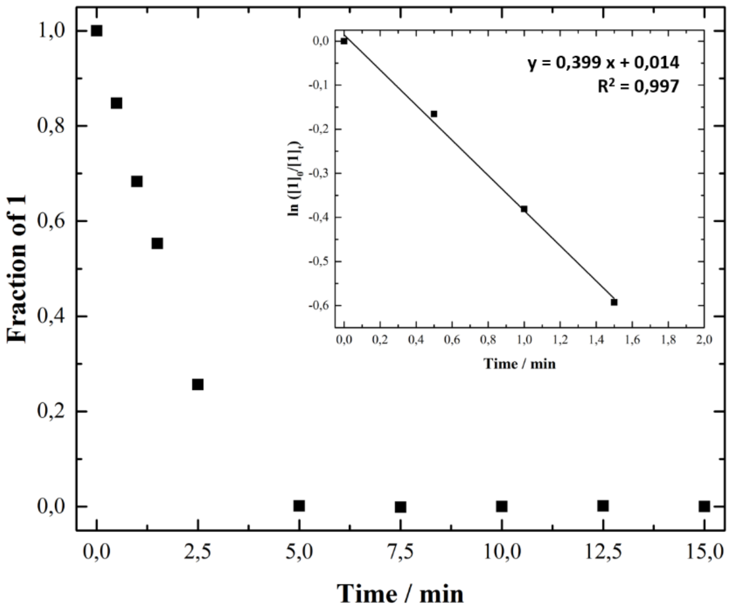

Supplementary Figure 9 | Photopolymerization kinetics of **1** in $CDCl_3$ (0.3 M, 18 °C) at 544 nm show a pseudo-first order reaction from which k_{obs} = 0.4 min^{-1} and $t_{1/2}$ = 1.7 min could be determined (see inset). The free monomer concentration as a function of time was determined via offline ^{1}H-NMR analysis in which all non-polymerized TAD was quantitatively converted into an irreversible Diels-Alder adduct by the addition of cyclopentadiene (1.2 eq.) upon irradiation.

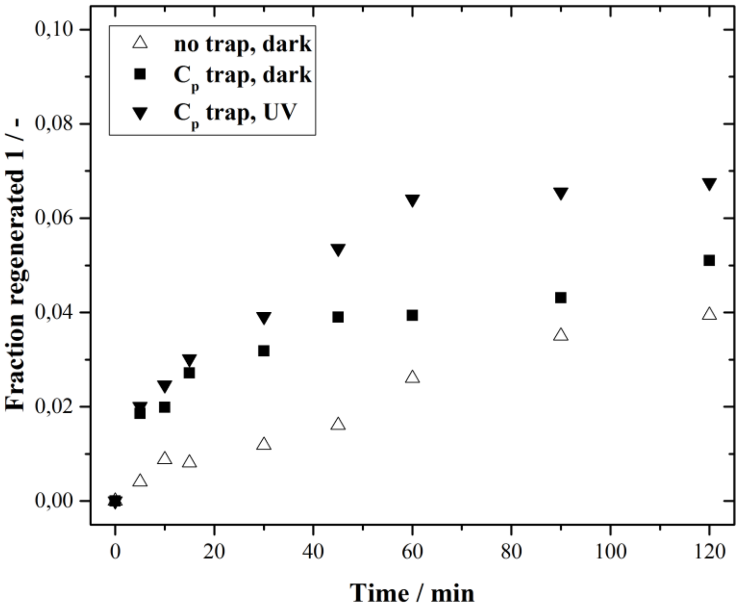

Supplementary Figure 10 | Fraction of regenerated **1** when kept in the dark after photodeactivation (open symbol). The presence of cyclopentadiene as an *in situ* trap for any released **1** only slightly increases depolymerization (closed symbol). The accelerated regeneration under UV-irradiation (λ_{max} = 320 nm, ARIMED B6, 3 x 36W) can be attributed to a higher temperature inside the photoreactor (25 °C) compared to the experiments carried out in the dark (18 °C) (cfr. Supplementary Fig. 11).

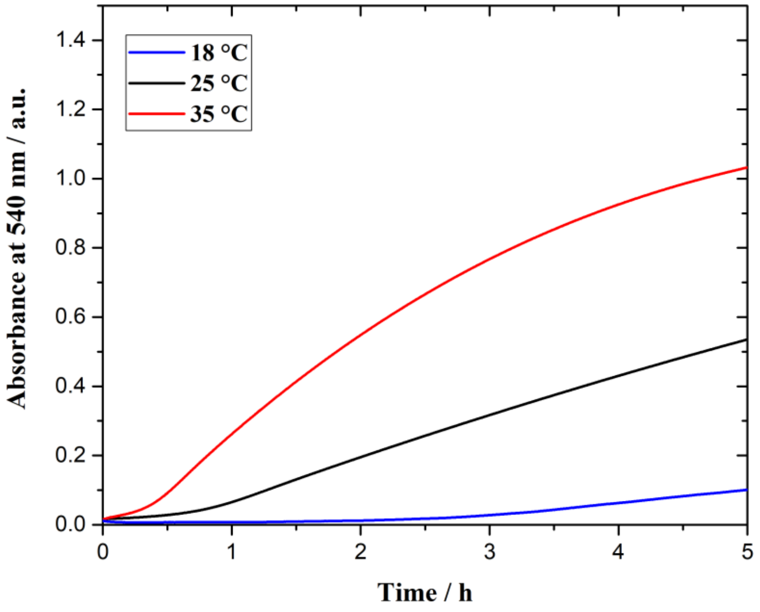

Supplementary Figure 11 | Kinetic UV/vis spectra ($CHCl_3$) recorded after 5 minutes of visible light irradiation (544 nm, 4 mW cm^{-2}, 100 Hz) of a 0.3 M solution of **1** in $CHCl_3$ showing the regeneration of **1** at different temperatures, i.e. 18 °C (blue), 25 °C (black) and 35 °C (red).

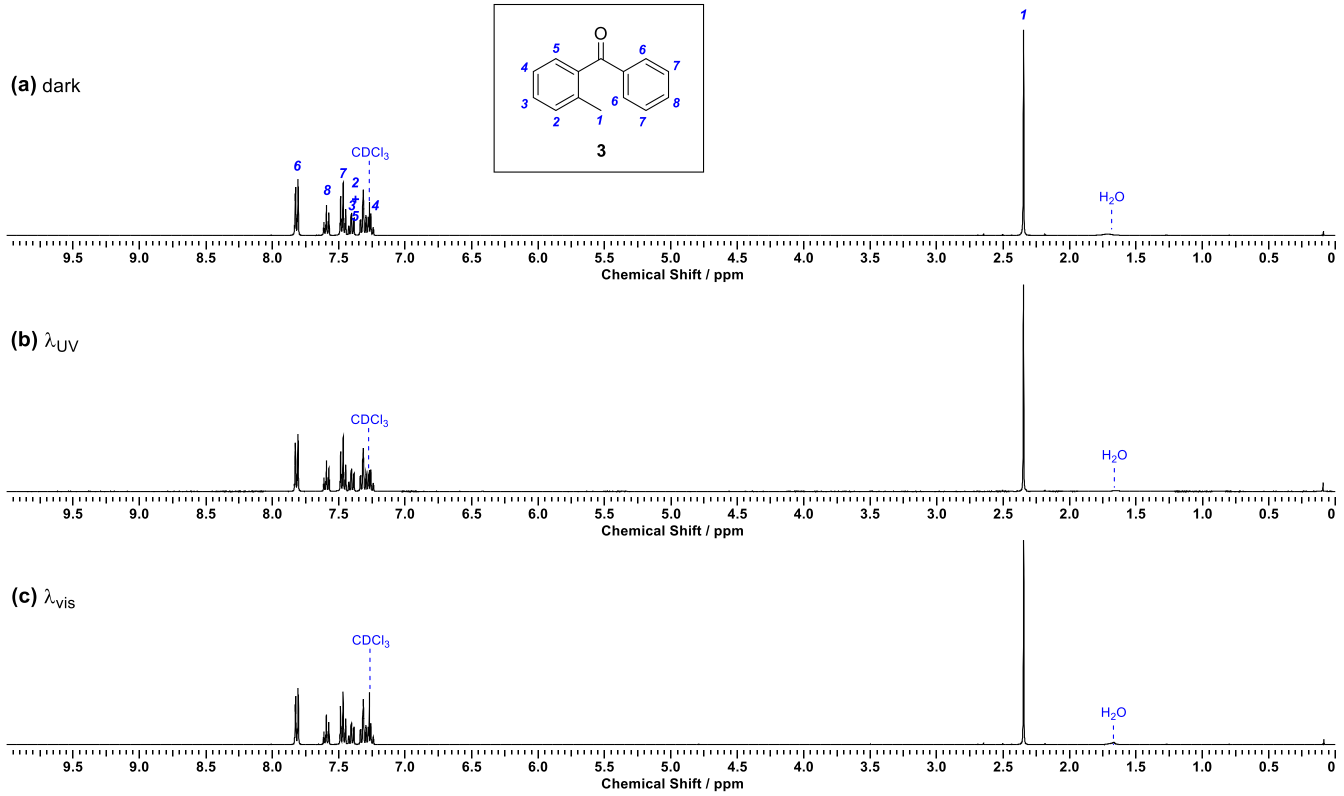

Supplementary Figure 12 | ^{1}H-NMR spectra of *o*-methylbenzophenone **3** (0.15 M, $CDCl_3$) before (a) and after irradiation with UV-light (λ_{max} = 320 nm, ARIMED B6, 3 x 36W, 4 h) (b) and visible light (544 nm, tunable laser, 4.0 mW cm^{-2}, 100 Hz, 1 h) (c), indicating no photodegradation.

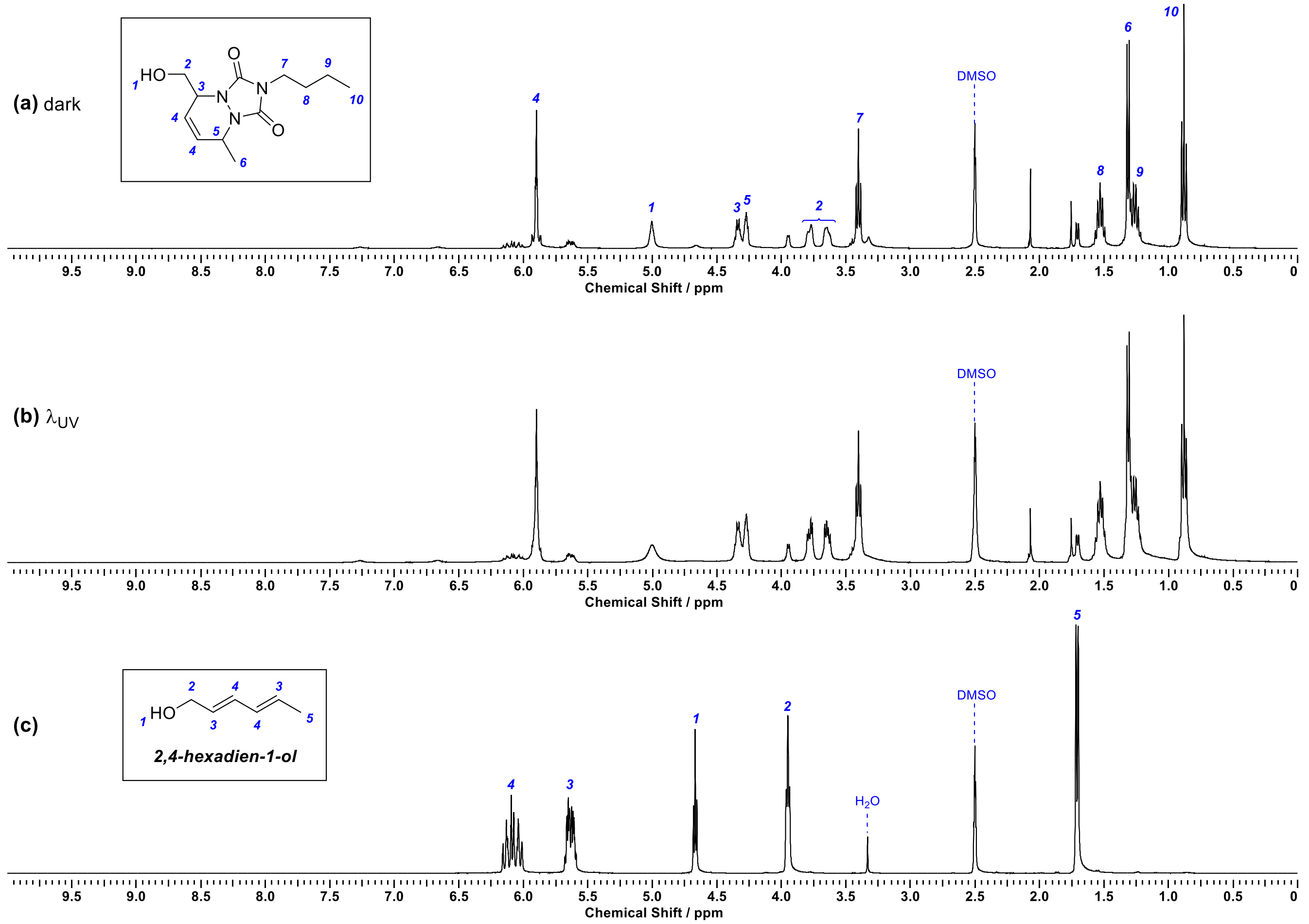

Supplementary Figure 13 | ^{1}H-NMR spectra (DMSO-d_6) of **1** after addition of *trans*,*trans*-2,4-hexadien-1-ol (1.1 eq) before (a) and after (b) irradiation with UV-light (λ_{max} = 320 nm, ARIMED B6, 3 x 36W, 4 h), indicating no photodegradation. Residual signals can be attributed to the slight excess of the diene trap (c). Spectra were measured in DMSO-d_6 to ensure better solubility of the potentially formed degradation products.

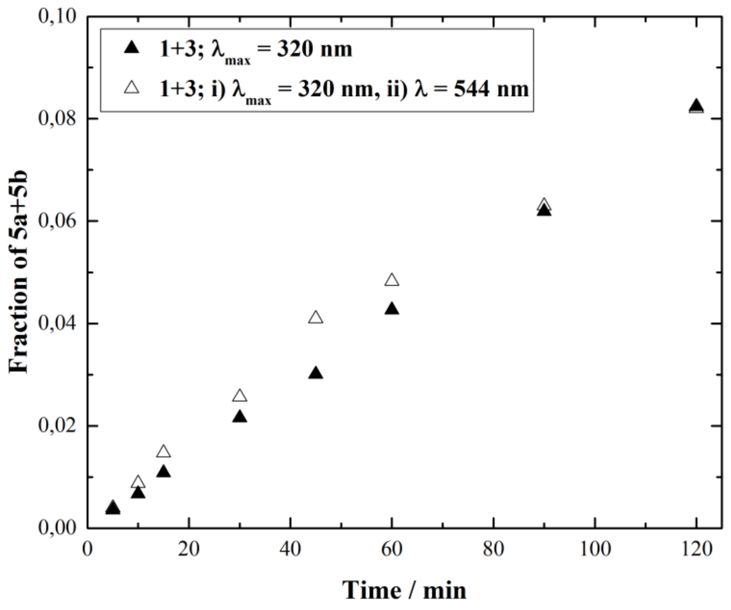

Supplementary Figure 14 | Fraction of reaction products **5a**+**5b** obtained from the photoinduced Diels-Alder reaction upon UV-light irradiation (λ_{max} = 320 nm, ARIMED B6, 3 x 36W) of an equimolar solution of **1** and **3** (0.15 M, $CDCl_3$). Similar conversions are obtained with (open symbol) and without (closed symbol) subsequent visible light-irradiation at 544 nm (tunable laser, 4.0 mW cm^{-2}, 100 Hz).

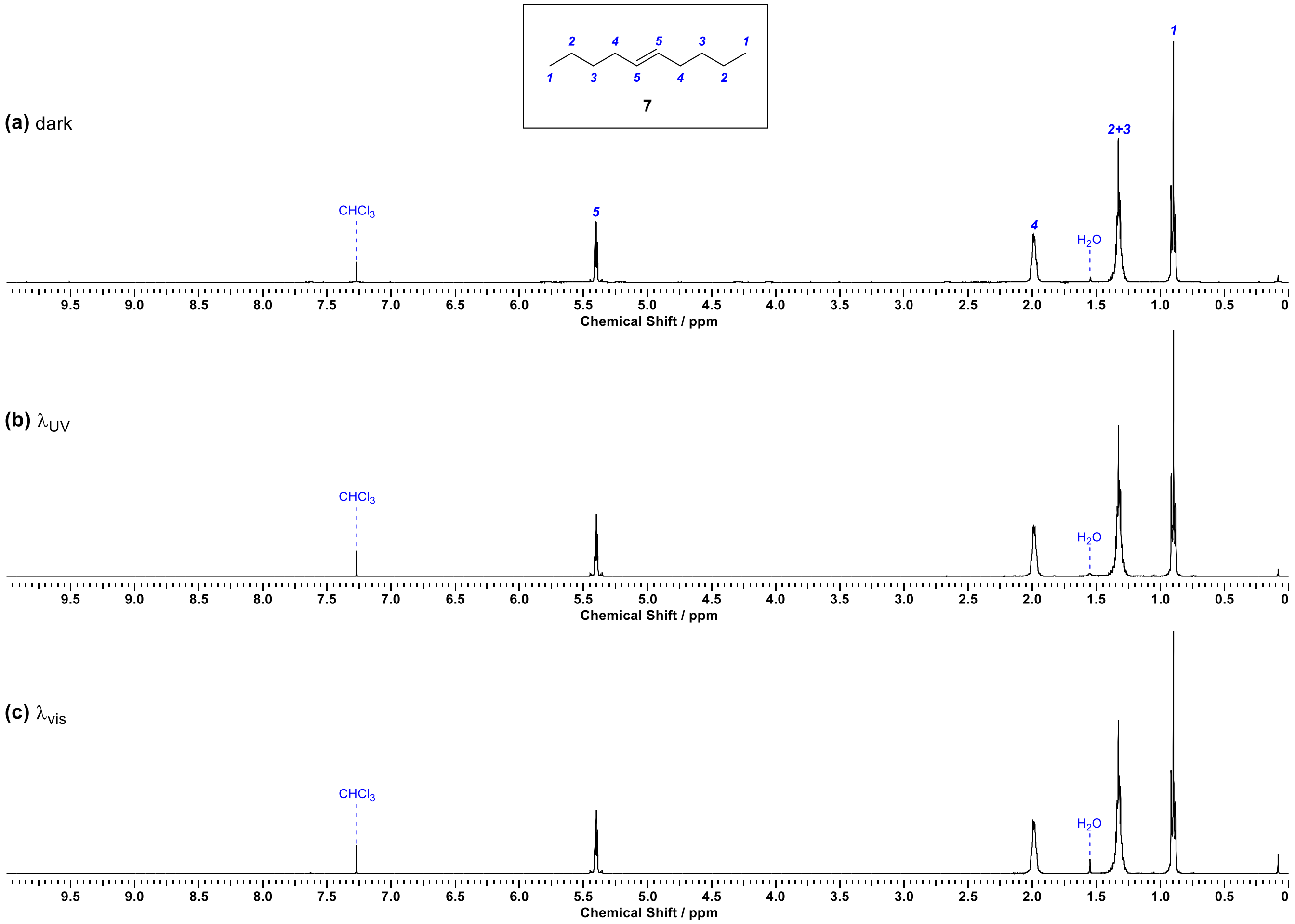

Supplementary Figure 15 | ^{1}H-NMR spectra of *trans*-5-decene **7** (0.15 M, $CDCl_3$) before (a) and after irradiation with UV-light (λ_{max} = 320 nm, ARIMED B6, 3 x 36W, 4 h) (b) and visible light (544 nm, tunable laser, 4.0 mW cm^{-2}, 100 Hz, 1 h) (c), indicating no photodegradation.

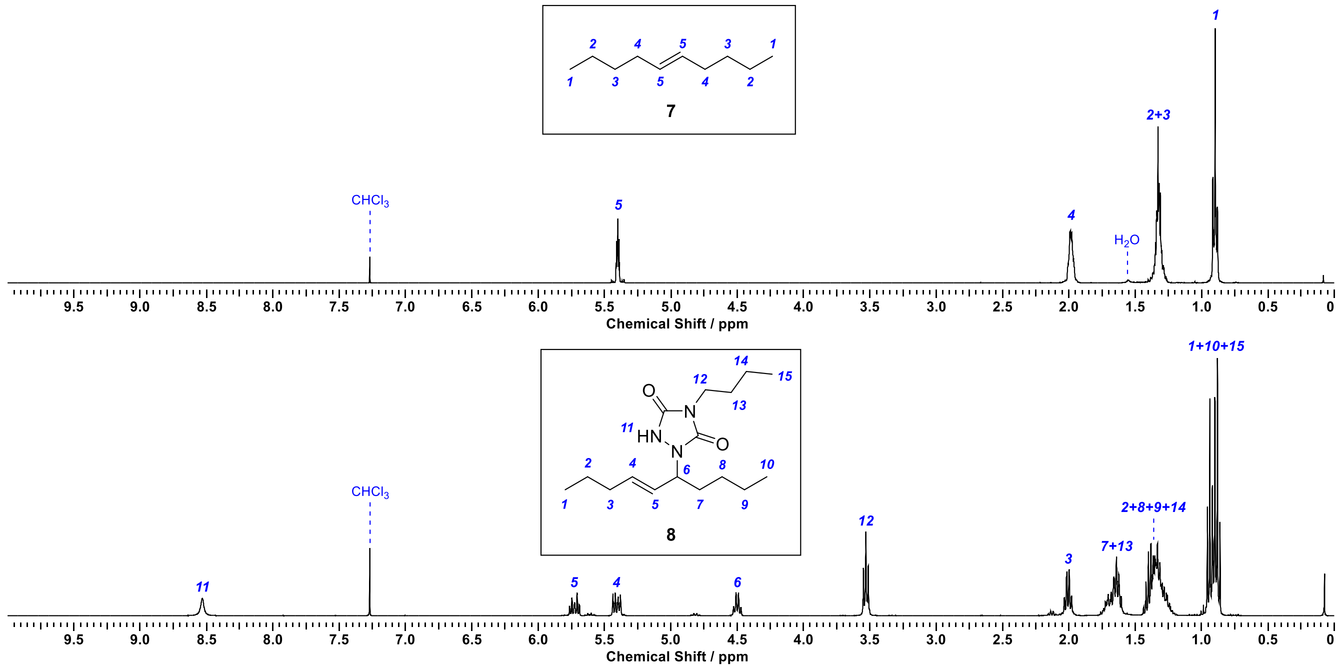

Supplementary Figure 16 | Exclusive formation of the 1:1 Alder-ene adduct **8** observed in the ^{1}H-NMR spectrum upon addition of 4-*n*-butyl-1,2,4-triazoline-3,5-dione **1** to *trans*-5-decene **7** (1:1 eq., $CDCl_3$, 0.15 M).

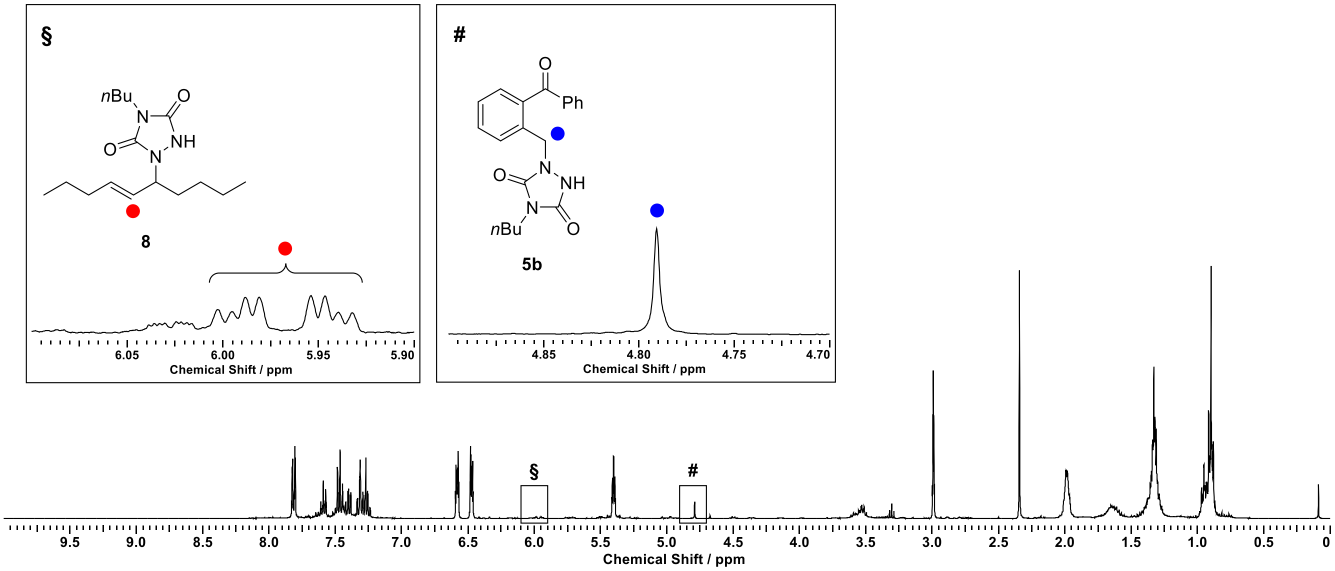

Supplementary Figure 17 | Representative ^{1}H-NMR spectrum of the reaction mixture of the switchable manifold (containing an equimolar mixture of photopolymer **2**, *o*-methylbenzophenone **3** and *trans*-5-decene **7**, 0.15 M in $CDCl_3$) after 2 h UV-irradiation, 8 h standing in the dark, 1 h irradiation with visible light, 4 h UV-irradiation and 8 h in the dark. The conversion of the photoproducts **5a**+**5b** and thermal TAD-addition product **8** were determined via integration of the well resolved signals (inset).

Supplementary Discussion

Structure elucidation of photoenol products 5a+5b

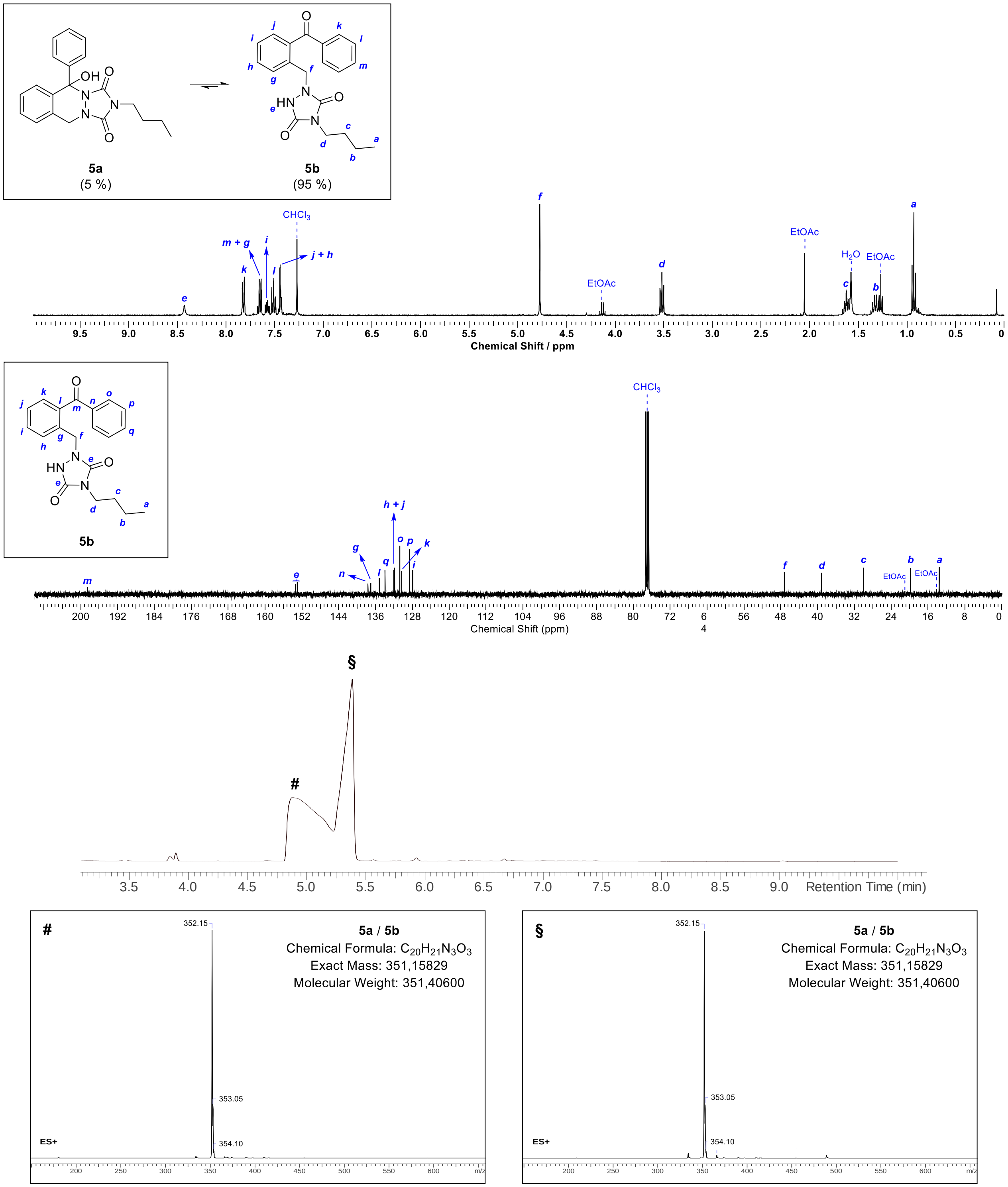

Supplementary Figure 18 | ^{1}H- and ^{13}C-NMR spectra ($CDCl_3$) and LC-MS trace with corresponding mass spectra used for the structure elucidation of the photoenol product mixture **5a**+**5b**. All peaks were assigned in agreement with 2D NMR spectra (i.e. HSQC, COSY and HMBC).

Structure elucidation of reduction product 6

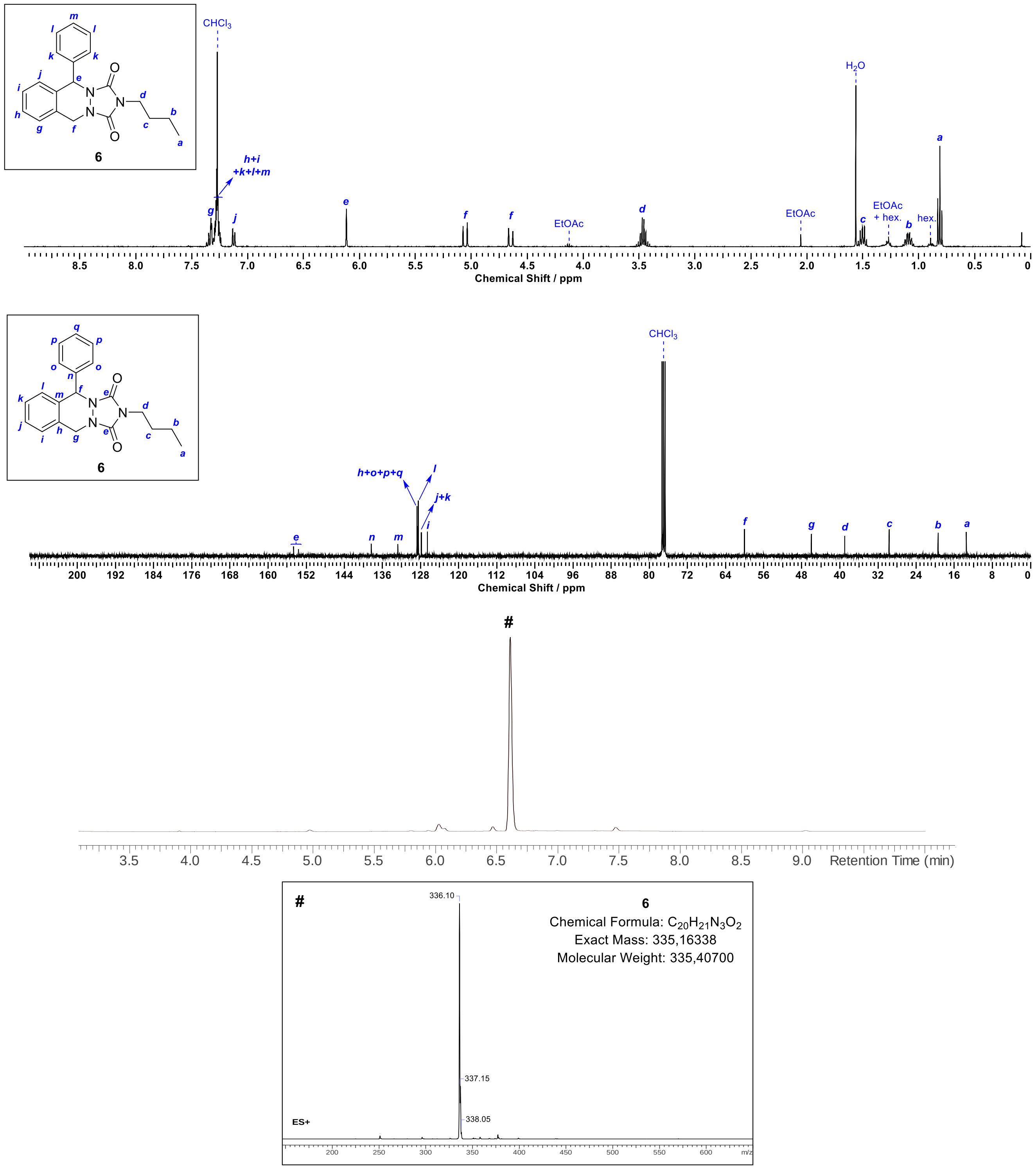

Supplementary Figure 19 | ^{1}H- and ^{13}C-NMR spectra ($CDCl_3$) and LC-MS trace with corresponding mass spectrum used for the structure elucidation of reduction product **6**. All peaks were assigned in agreement with 2D NMR spectra (i.e. HSQC, COSY and HMBC).

Structure elucidation of the 1:2 adduct of 1 and 3

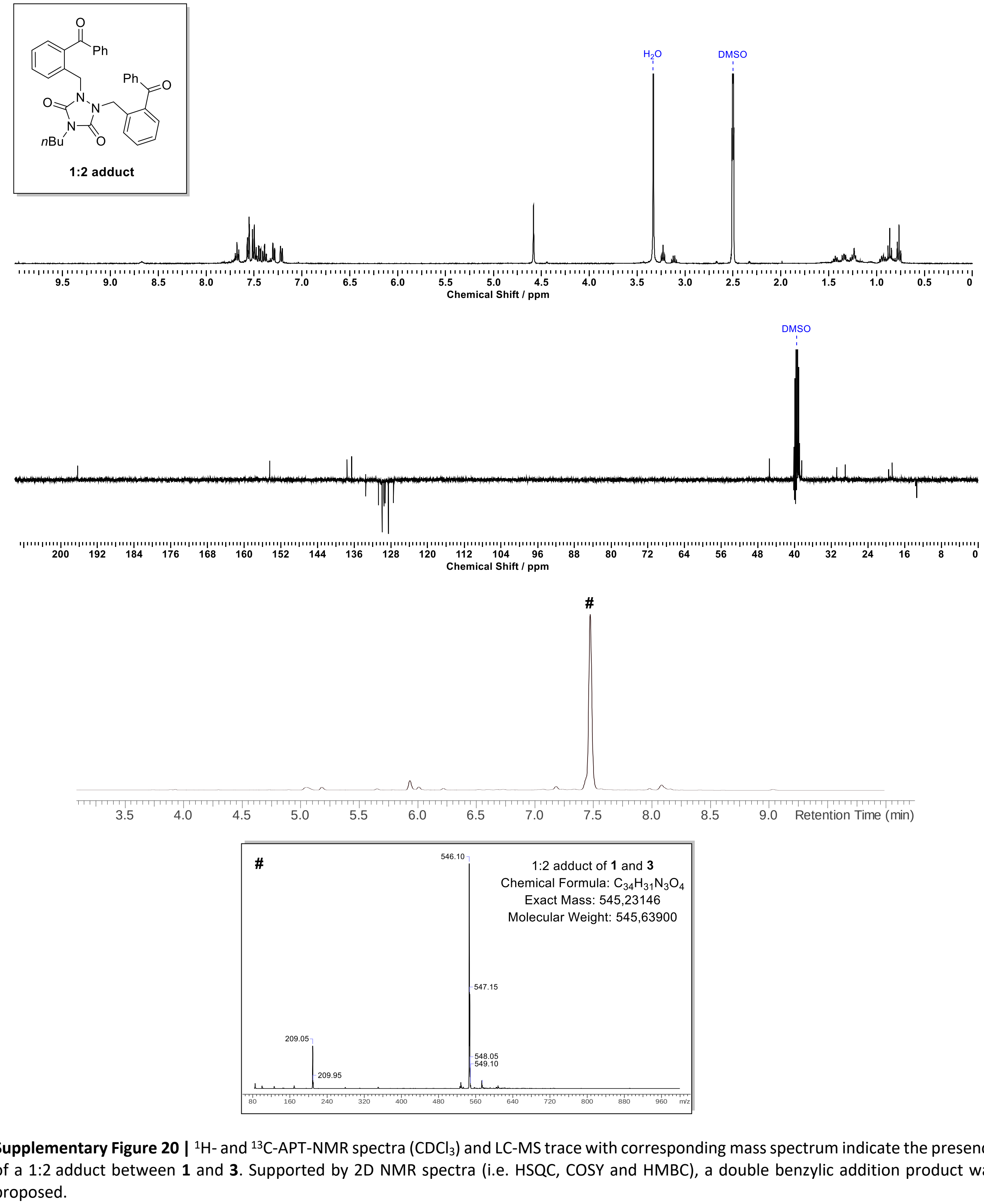

Supplementary Figure 20 | ^{1}H- and ^{13}C-APT-NMR spectra ($CDCl_3$) and LC-MS trace with corresponding mass spectrum indicate the presence of a 1:2 adduct between **1** and **3**. Supported by 2D NMR spectra (i.e. HSQC, COSY and HMBC), a double benzylic addition product was proposed.

Supplementary Methods

Instrumentation

Custom-built photoreactor. Irradiation experiments with ARIMED B6 (3 x 36W) compact fluorescent lamps (λ_{max} = 320 nm) were carried out in a custom-built photoreactor (refer to Supplementary Figure 21). To ensure efficient light penetration, the samples were placed in a circular sample holder, which is rotated around the central lamp with an angular velocity of 0.5 rad s^{-1}. The temperature inside the photoreactor was continuously monitored, but did not exceed 25 °C. All samples subjected to UV-irradiation were prepared in 20 mL headspace vials (clear glass, rounded bottom, long neck; VWR international, Art. Nr. 548-0891) and crimped air-tight with aluminium caps with septa (central hole 10 mm; VWR international, Art. Nr. 548-0060). For sample preparation, we refer to the experimental section.

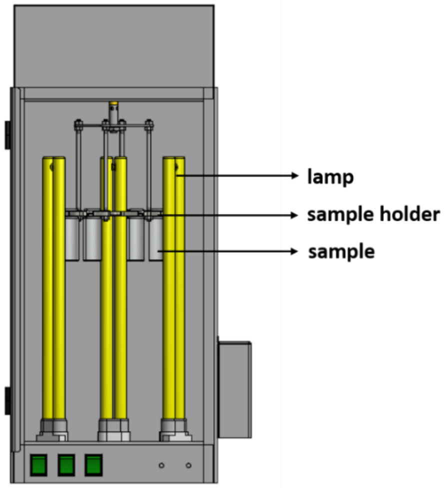

Supplementary Figure 21 | Schematic representation of the custom-built photoreactor.

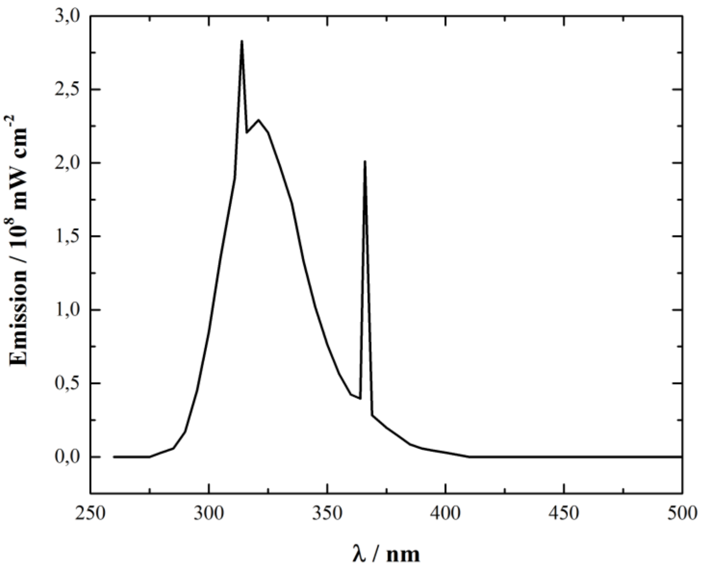

Supplementary Figure 22 | Emission spectrum of the ARIMED B6 lamp (λ_{max} = 320 nm, 36 W) used for UV-light irradiation experiments.

Electrospray Ionization Mass Spectrometry (ESI-MS). Mass spectra were recorded on a LTQ Orbitrap XL Q Exactive mass spectrometer from ThermoFischer Scientific, equipped with a HESI II probe and calibrated using premixed calibration standards containing caffeine, Met-Arg-Ph-Ala acetate and a mixture of fluorinated phosphazenes in an m/z = 74 – 1822 range. Samples were dissolved in a THF:MeOH mixture (HPLC grade) doped with 100 µmol sodium trifluoroacetate (NaTFA) and injected with a flow rate of 5 µL min^{-1} at a constant spray voltage of 4.6 kV, capillary temperature of 320 °C and S-lens RF level set to 62.0.

Hyphenated Liquid Chromatography – Mass Spectrometry (LC-MS). LC-MS measurements were performed on an Agilent Technologies 1100 series LC/MSD system containing a diode array and single quad MS detector coupled to an electrospray ionization source (ESI-MS). Analytical reversed phase HPLC was carried out on a Phenomex Luna C18 (2) column (dimensions 5 µm x 250 mm x 4.6 mm) in a solvent mixture of acetonitrile in water using a gradient of 0 to 100 % in 15 minutes and the eluted compounds were detected at 214 nm or 254 nm.

High resolution mass spectrometry was carried out with an Agilent 6220 accurate-mass time-of-flight (TOF) containing a multimode ionization (MMI) source.

Analysis was performed using the ACD/Labs Spectrus software.

Tunable Laser System. Wavelength-tunable UV- and visible laser light was generated with an Innolas Splitlight 600 OPO Nd:YAG Tunable Laser System using an Optical Parametric Oscillator (OPO) to produce a tunable output between 410 and 670 nm or, with a second modular unit, between 270 and 410 nm. The OPO is operated by a diode pumped Nd:YAG laser with a 100 Hz repetition rate. The output energy was regulated by a variable attenuator (polarizer) coupled to an Energy Max PC power meter (Coherent) to measure the energy of the incident laser pulses. The number of pulses was chosen freely and can directly be correlated to the total irradiation time. The generated laser light was guided through a prism to irradiate the bottom of the sample which was placed in a home-made sample holder (refer to Supplementary Figure 1). The temperature in the laser room was regulated at 18 °C.

All samples subjected to laser irradiation were prepared in 0.7 mL crimp neck vials (clear glass, flat bottom; LLG Labware, Lab Logistic Group GmbH, Art. Nr. 4008202) sealed air-tight with aluminium caps with septa (central hole 4 mm; VWR international, Art. Nr. 548-0038).

For sample preparation, we refer to the experimental procedures section.

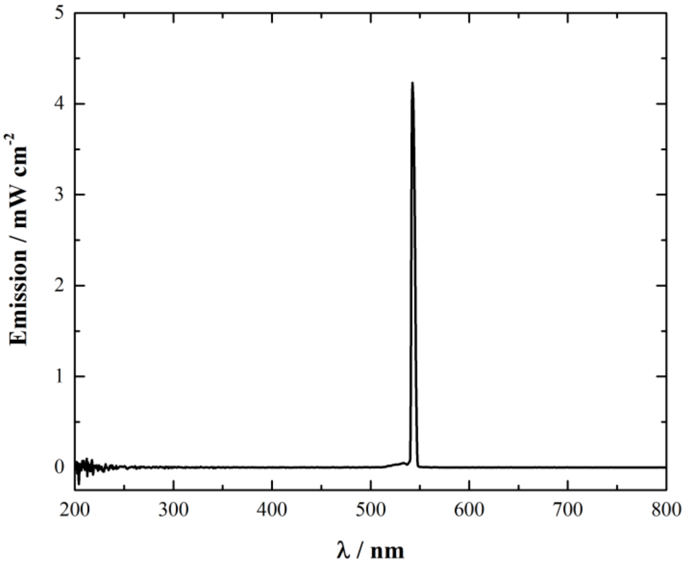

Supplementary Figure 23 | Emission spectrum of the wavelength-tunable laser set at 544 nm (2.0 mJ, 100 Hz), used for the visible light irradiation experiments.

Nuclear Magnetic Resonance (NMR) Spectrometry. NMR spectra were recorded on a Bruker Avance 300 (300 MHz), Bruker Ascend 400 (400 MHz) or Bruker Avance II (500 MHz) FT-NMR spectrometer at room temperature in the solvent as indicated. Chemical shifts δ are expressed in parts per million (ppm) with the residual solvent peaks taken as an internal standard. The resonance multiplicities are abbreviated as follows: s (singlet), d (doublet), t (triplet), q (quadruplet), quint (quintuplet), sext (sextuplet) or m (multiplet).

UV/vis Emission Spectrometer. Emission spectra of irradiation sources were recorded with a UV/vis SR600 spectrometer containing a polychromator and a silicon photodiode array. The device is calibrated to enable accurate spectral radiometric measurements, which are processed with the SR600 spectral software. A dark measurement was carried out prior to each calibrated measurement and the integration time was changed appropriately to obtain a recording level of approximately 90%.

UV/vis Spectrometry. UV/vis spectra were recorded on a Varian Cary 300 Bio spectrometer at 25 °C and on a Shimadzu UV-2700 spectrophotometer coupled to a CPS-100 cell positioner for thermoelectrically temperature controlled measurements.

Materials

Anhydrous acetonitrile (99.8 %, Acros Organics); carbon tetrachloride (Acros Organics); chloroform (> 99.5 %, VWR); *trans*-5-decene (99 %, Sigma-Aldrich); deuterated chloroform ($CDCl_3$, Aldrich); deuterated dimethyl sulfoxide (DMSO-d_6, EurisoTOP); dicyclopentadiene (99 %, abcr GmbH); *N*-ethylmaleimide (crystalline, > 98 %, Sigma-Aldrich); 2-methylbenzophenone (98 %, Sigma-Aldrich); *trans*,*trans*-2,4-hexadien-1-ol (> 97 %, Sigma-Aldrich); triethylsilane (97 %, Sigma-Aldrich); trifluoroacetic acid (99 %, VWR).

All solvents and products were used as received from their supplier.

4-*n*-butyl-1,2,4-triazoline-3,5-dione (**1**) was synthesized according to a literature procedure,[3] followed by sublimation at 40 °C under reduced pressure (10^{-1} mbar). The resulting purple crystalline product was stored in the dark at -18°C and used within two weeks.

Cyclopentadiene (**C_p**) was obtained as a colorless liquid by thermal cracking of dicyclopentadiene at 180 °C, which was stored at -18 °C for up to 3 days.

Experimental Procedures

Photopolymerization of 1 upon visible light irradiation

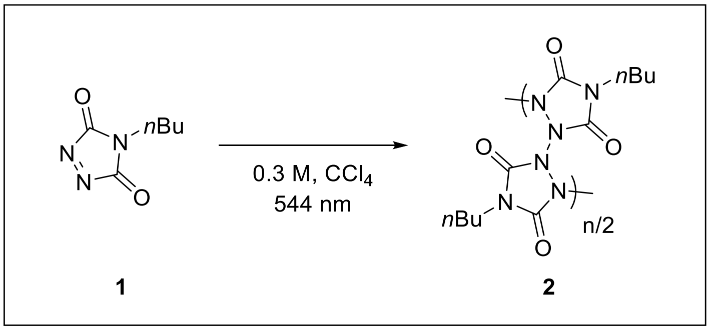

Supplementary Figure 24 | Photopolymerization of **1** upon visible light irradiation.

A 0.3 M stock solution of **1** (69.8 mg, 0.60 mmol) in carbon tetrachloride (1.5 mL) was sealed with a septum and deoxygenated by flushing with $N_{2\,(g)}$ for 15 minutes. The stock solution was next divided into portions of 0.2 mL in crimp neck vials (0.7 mL) which were quickly flushed with $N_{2\,(g)}$ and crimped air-tight. The bright purple solution was then placed in the wavelength-tunable laser sample holder and irradiated at 544 nm (4.0 mW cm^{-2}, 100 Hz) to give a clear colorless solution of photopolymer **2** after 10 minutes.

Upon standing in the dark, the reaction mixture regained a faint purple color (refer to Supplementary Figure 2) which was evidenced via UV/vis kinetic measurements of a diluted solution (1 mM) at λ_{max} = 544 nm (refer to Supplementary Figure 3).

The photopolymerization was also successfully carried out at lower wavelengths (i.e. down to 440 nm) – keeping the number of incident photons constants (*vide infra*) – albeit with longer irradiation times (up to 30 min).

Similar results were obtained upon changing the solvent to deuterated chloroform, enabling ^{1}H-NMR analysis (cfr. Supplementary Figure 4). **^{1}H-NMR (400 MHz, $CDCl_3$):** after 8 minutes of dark time, δ (ppm) = 0.95 (broad m, 3H, CH_3), 1.40 (broad m, 3H, CH_3-CH_2), 1.68 (broad m, 3H, N-CH_2-CH_2), 3.31 + 3.60 (t + broad m, 2H, N-CH_2).

In acetonitrile, solutions of **1** did not give a clear colorless solution, even after prolonged irradiation times of 1 h (refer to Supplementary Figure 7). Furthermore, no increase in absorbance was observed over time.

Photostability assessment of 1 as a function of the applied wavelength

An air-tight crimped headspace vial with septum containing **1** (36 mg, 0.23 mmol) was placed under $N_{2\,(g)}$ atmosphere. To this, 7.2 mL of deoxygenated (by flushing with $N_{2\,(g)}$ for 20 min) anhydrous acetonitrile was added. The resulting purple stock solution was divided into 0.25 mL portions in crimp neck vials (0.7 mL) which were quickly flushed with $N_{2\,(g)}$ and crimped air-tight. Each sample was placed in the wavelength-tunable laser sample holder and irradiated at a distinct wavelength, ranging from 320 to 560 nm with 10 nm intervals. The target pulse energy of the laser was set carefully to ensure for a constant number of incident photons hitting the sample, independent of the wavelength applied (refer to Supplementary Table 3). This target energy was calculated according to a calibration curve reported elsewhere,[1, 2] yet for the same laser system used in this study, in which a correction is made with regard to the absorption of the incident laser light by the glass of the sample recipient. The total number of incident photons was altered by changing the number of pulses emitted by the laser, which is directly proportional to the irradiation time. After irradiation, 0.1 mL of the resulting solution was diluted with 3.4 mL of acetonitrile and subjected to UV/vis analysis. The photostability was determined by the ratio of the absorbance (at λ_{max} = 527 nm) of the sample after irradiation to a reference sample kept in the dark. The results are depicted in Supplementary Figure 8.

Supplementary Table 3 | Parameters used for the photostability assessment of **1** in acetonitrile (5 mg mL^{-1}) at different wavelengths (from 320 to 560 nm with 10 nm intervals) for three different numbers of incident photons. The target pulse energy was calculated taking into account the absorbance of the glass recipient (by means of a calibration curve[1, 2]) to ensure for a constant number of photons deposited into the sample throughout the entire wavelength regime. N_p = number of incident photons.

**λ (nm)**	**Target pulse energy (µJ)**	**Measured pulse energy (µJ)**		
		$N_p = 3.42 .10^{18}$	$N_p = 8.54 .10^{18}$	$N_p = 1.71 .10^{19}$
**320**	627.2	628	628	628
**330**	562.1	567	563	562
**340**	537.3	537	535	536
**350**	500.0	502	499	502
**360**	471.2	470	472	470
**370**	470.3	469	470	470
**380**	454.7	456	454	454
**390**	428.9	429	430	429
**400**	422.5	421	427	423
**410**	384.4	385	385	385
**420**	366.2	365	366	366
**430**	349.3	349	348	349
**440**	338.6	338	340	337
**450**	327.9	329	328	327
**460**	318.6	323	314	315
**470**	310.3	309	308	310
**480**	300.5	298	299	301
**490**	291.8	293	290	293
**500**	284.0	286	284	285
**510**	276.3	278	277	278
**520**	268.9	270	271	268
**530**	261.8	262	262	261
**540**	255.1	254	253	254
**550**	248.5	250	248	250
**560**	242.3	242	243	241

Trapping experiment to demonstrate the photodeactivation of 1 upon visible light irradiation

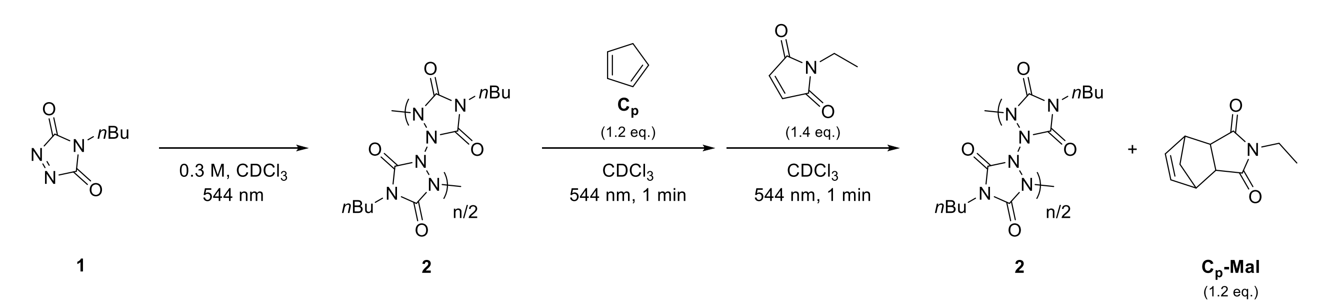

Supplementary Figure 25 | Trapping experiment upon visible light irradiation designed to demonstrate the photodeactivation of **1**.

An air-tight crimped headspace vial with septum containing **1** (93.1 mg, 0.60 mmol) was placed under $N_{2\,(g)}$ atmosphere. To this, 2.0 mL of deoxygenated (by flushing with $N_{2\,(g)}$ for 20 min) deuterated chloroform was added. 0.2 mL of the resulting 0.3 M stock solution of **1** (9.31 mg, 0.060 mmol, 1 eq.) was next transferred into a crimp neck vial (0.7 mL) and quickly flushed with $N_{2\,(g)}$ before crimped air-tight. The sample was placed in the wavelength-tunable laser sample holder and irradiated at 544 nm (4.0 mW cm^{-2}, 100 Hz) for 10 min to give a clear colorless solution of **2**. To this, under continuous irradiation with visible laser light, 0.2 mL of a 0.3 M solution of cyclopentadiene (**C_p**, 4.76 mg, 0.072 mmol, 1.2 eq.) was added through the septum of the crimp neck vial. Whilst the visible light is still kept switched on, the mixture was allowed to stand for 1 minute to ensure for any active TAD to react with **C_p**. Next, *N*-ethylmaleimide (10.5 mg, 0.084 mmol, 1.4 eq.) in 0.2 mL deuterated chloroform was added and allowed to react for 1 additional minute to quench any non-reacted **C_p** in the **C_p-Mal** Diels-Alder adduct before the visible light is finally switched off. The resulting mixture quickly regained a faint purple color due to the regeneration of **1**. Offline ^{1}H-NMR analysis (cfr. Figure 2) indicated no formation of the **C_p-1** Diels-Alder adduct and thus no active TAD species are present upon visible light irradiation, thereby demonstrating the quantititave photodeactivation of **1**.

Kinetic study of the photopolymerization of 1

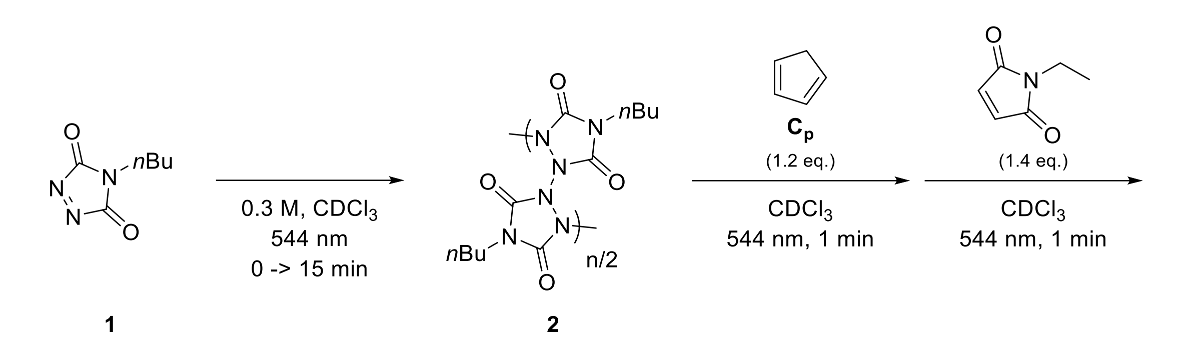

Supplementary Figure 26 | Kinetic study of the photopolymerization reaction of **1** upon visible light irradiation.

An air-tight crimped headspace vial with septum containing **1** (93.1 mg, 0.60 mmol) was placed under $N_{2\,(g)}$ atmosphere. To this, 2.0 mL of deoxygenated (by flushing with $N_{2\,(g)}$ for 20 min) deuterated chloroform was added. The resulting 0.3 M stock solution of **1** was divided into portions of 0.2 mL over several crimp neck vials (0.7 mL), quickly flushed with $N_{2\,(g)}$ and crimped air-tight. The solutions of **1** (9.31 mg, 0.060 mmol, 1 eq.) were subsequently placed in the wavelength-tunable laser sample holder and irradiated at 544 nm (4.0 mW cm^{-2}, 100 Hz). Each sample was subjected to visible light for a well-defined time period (i.e. 0; 0.5; 1.0; 1.5; 2.5; 5.0; 7.5; 10.0; 12.5 and 15.0 min). The fraction of remaining monomer **1** was locked-in by the addition of 0.2 mL of a 0.3 M solution of cyclopentadiene (**C_p**, 4.76 mg, 0.072 mmol, 1.2 eq.) after a certain time of irradiation. The mixture was allowed to stand for 1 minute in order to trap all remaining TAD into the **C_p-1** Diels-Alder adduct, whilst the visible light is still kept switched on. Next, *N*-ethylmaleimide (10.5 mg, 0.084 mmol, 1.4 eq.) in 0.2 mL deuterated chloroform was added and allowed to react for 1 additional minute to quench the excess of **C_p** in a second **C_p-Mal** Diels-Alder adduct before the visible light is finally switched off. The obtained reaction mixtures were subjected to offline ^{1}H-NMR analysis from which the monomer conversion over time, depicted in Supplementary Figure 9, was determined.

Regeneration kinetics of 1 after photopolymerization

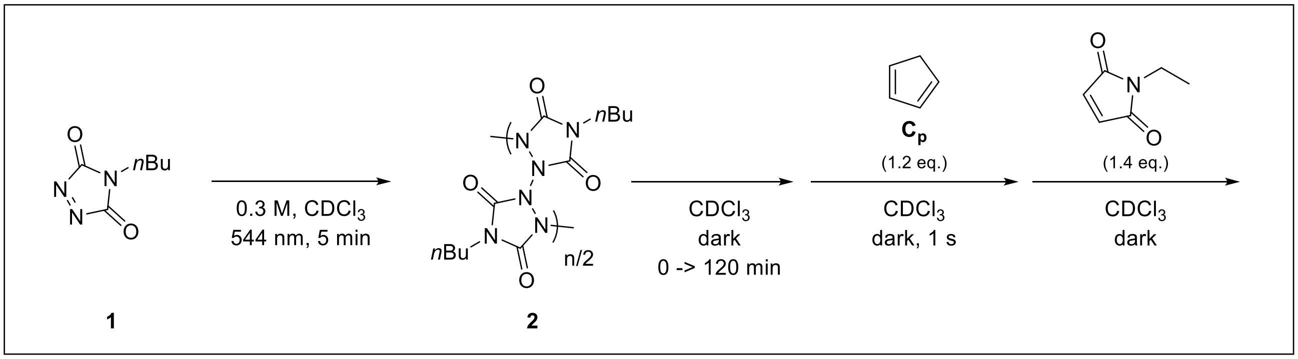

Supplementary Figure 27 | Investigation of the regeneration kinetics of 1 after photopolymerization upon visible light irradiation.

An air-tight crimped headspace vial with septum containing **1** (93.1 mg, 0.60 mmol) was placed under $N_{2\,(g)}$ atmosphere. To this, 2.0 mL of deoxygenated (by flushing with $N_{2\,(g)}$ for 20 min) deuterated chloroform was added. The resulting 0.3 M stock solution of **1** was divided into portions of 0.2 mL over several crimp neck vials (0.7 mL), quickly flushed with $N_{2\,(g)}$ and crimped air-tight. The solutions of **1** (9.31 mg, 0.060 mmol, 1 eq.) were next placed in the wavelength-tunable laser sample holder and irradiated at 544 nm (4.0 mW cm^{-2}, 100 Hz) for 5 minutes to give a clear colorless solution of **2**. Each sample was allowed to stand in the dark (18 °C) for a well-defined time period (i.e. 0; 5; 10; 15; 30; 45; 60; 90 and 120 min) to regenerate the initial purple colored monomer. The fraction of regenerated **1** was then locked-in by the addition of 0.2 mL of a 0.3 M solution of cyclopentadiene (**C_p**, 4.76 mg, 0.072 mmol, 1.2 eq.) after a certain dark time to form the **C_p-1** Diels-Alder adduct. After 1 s, *N*-ethylmaleimide (10.5 mg, 0.084 mmol, 1.4 eq.) in 0.2 mL deuterated chloroform was added to quench the excess of **C_p** trap in a second **C_p-Mal** Diels-Alder adduct. The obtained reaction mixtures rapidly regained a faint purple color caused by the continued release of **1** over time and were subjected to offline ^{1}H-NMR analysis from which the fraction of regenerated **1** was determined over time (refer to Supplementary Figure 10).

The same experiment was repeated, yet with the addition of **C_p** immediately after the visible light induced photopolymerization in order to determine the regeneration of **1** in the presence of **C_p** as an *in situ* trap (refer to Supplementary Figure 10). In this case, the fraction of regenerated **1** was locked-in by the addition of *N*-ethylmaleimide after standing in the dark (18 °C) for a well-defined time period (i.e. 0; 5; 10; 15; 30; 45; 60; 90 and 120 min). Alternatively, the exact same regeneration experiment, i.e. with the addition of **C_p** immediately after the visible light induced photopolymerization, was also carried out upon UV-irradiation at λ_{max} = 320 nm (3 x 36 W ARIMED B6 lamps) in the custom-built photoreactor (25 °C) (refer to Supplementary Figure 10).

The regeneration kinetics at different temperatures (i.e. 18 °C, 25 °C and 35 °C) were monitored for 5 h at λ_{max} = 540 nm in a UV/vis spectrophotometer coupled to a temperature control unit. For this, the solution of **2** after visible light irradiation was diluted with chloroform to a concentration of 7.5 mM before transferred into a cuvette (refer to Supplementary Figure 11).

Photostability assessment of *o*-methylbenzophenone 3 and *trans*-5-decene 7

Upon UV-light irradiation

An air-tight crimped headspace vial with septum containing *o*-methylbenzophenone **3** (29.4 mg, 0.15 mmol) and/or *trans*-5-decene **7** (21.0 mg, 0.15 mmol) was placed under $N_{2\,(g)}$ atmosphere. To this, 1.0 mL of deoxygenated (by flushing with $N_{2\,(g)}$ for 20 min) deuterated chloroform was added. The resulting 0.15 M solution was transferred into a custom-built photoreactor and irradiated with ARIMED B6 compact fluorescent lamps (3 x 36 W, λ_{max} = 320 nm) for 4 h. The photostability was determined by comparison of the ^{1}H-NMR spectra before and after irradiation (refer to Supplementary Figure 12 and Supplementary Figure 15).

Upon visible light irradiation

An air-tight crimped headspace vial with septum containing *o*-methylbenzophenone **3** (29.4 mg, 0.15 mmol) and/or *trans*-5-decene **7** (21.0 mg, 0.15 mmol) was placed under $N_{2\,(g)}$ atmosphere. To this, 1.0 mL of deoxygenated (by flushing with $N_{2\,(g)}$ for 20 min) deuterated chloroform was added. 0.4 mL of the resulting 0.15 M solution was transferred into a crimp neck vial (0.7 mL) and quickly flushed with $N_{2\,(g)}$ before crimped air-tight. The sample was placed in the wavelength-tunable laser sample holder and irradiated at 544 nm (4.0 mW cm^{-2}, 100 Hz) for 1 h. The photostability was determined by comparison of the ^{1}H-NMR spectra before and after irradiation (refer to Supplementary Figure 12 and Supplementary Figure 15).

Photostability assessment of 1 upon irradiation with ARIMED B6 UV-lamps

An air-tight crimped headspace vial with septum containing 4-*n*-butyl-1,2,4-triazoline-3,5-dione **1** (9.31 mg, 0.06 mmol, 1.0 eq.) was placed under $N_{2\,(g)}$ atmosphere. To this, 0.2 mL of deoxygenated (by flushing with $N_{2\,(g)}$ for 20 min) deuterated chloroform was added. The resulting 0.3 M solution was transferred into a custom-built photoreactor and irradiated with ARIMED B6 compact fluorescent lamps (3 x 36 W, λ_{max} = 320 nm) for 4 h. Immediately after irradiation, a slight excess of *trans*,*trans*-2,4-hexadien-1-ol (6.5 mg, 0.07 mmol, 1.1 eq.) in 0.3 mL deuterated chloroform was added. The photostability was determined by comparison of the ^{1}H-NMR spectra after irradiation with a dark non-irradiated sample (refer to Supplementary Figure 12).

Light-induced photoenol reaction of 1 with 3

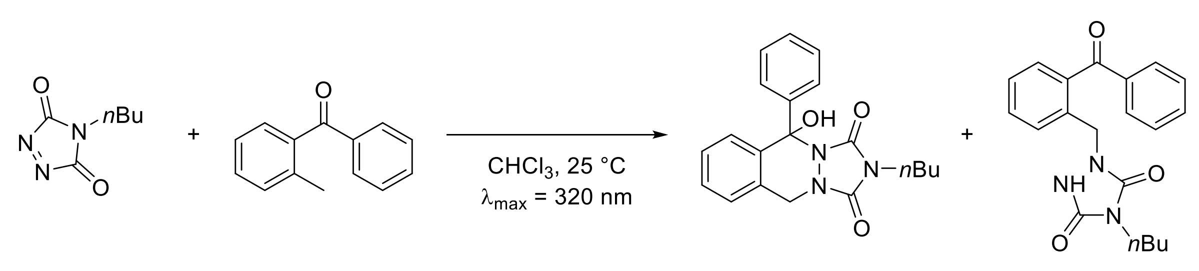

Supplementary Figure 28 | UV-light-induced photoenol reaction of **1** with **3**.

A mixture of 4-*n*-butyl-1,2,4-triazoline-3,5-dione **1** (93.1 mg, 0.6 mmol, 1.0 eq.) and *o*-methylbenzophenone **3** (118 mg, 0.6 mmol, 1.0 eq.) in 20 mL of deoxygenated chloroform (by flushing with $N_{2\,(g)}$ for 10 min) was added to an air-tight capped headspace crimped vial placed under nitrogen atmosphere. The mixture was placed in a custom-built photoreactor and irradiated with 3 x 36 W ARIMED B6 compact fluorescent lamps (λ_{max} = 320 nm) to give a clear faint yellow solution after 2.5 h. Solvent removal *in vacuo* yielded a yellow oil containing an equilibrium mixture of **5a** and **5b**. Purification via column chromatography (silica) failed to result in separation of the product mixture (156 mg – 74 %). **5a**:**5b** = 1:14 in $CDCl_3$; **5a**:**5b** = 1:3 in DMSO-d_6.

^{1}H-NMR (500 MHz, $CDCl_3$): 5b, δ (ppm) = 0.93 (t, 3H, CH_3), 1.32 (m, 2H, CH_3-CH_2), 1.62 (m, 2H, N-CH_2-CH_2), 3.52 (t, 3H, N-CH_2), 4.77 (s, 2H, Ar-CH_2), 7.40-7.47 (m, 2H, Ar*H*), 7.48-7.54 (t, 2H, Ar*H*), 7.55-7.60 (m, 1H, Ar*H*), 7.62-7.69 (m, 2H, Ar*H*), 7.82 (m, 2H, Ar*H*), 8.43 (s, 1H, N*H*); **5a**, some resolved resonances: 4.80 (d, 1H, CH_2), 4.96 (d, 1H, CH_2). **^{13}C-NMR (500 MHz, $CDCl_3$):** δ (ppm) = 13.58 (CH_3), 19.81 (CH_2), 30.02 (CH_2), 39.12 (CH_2), 47.19 (CH_2), 127.91 (CH), 128.61 (CH), 130.30 (CH), 130.74 (CH), 131.86 (CH), 132.02 (CH), 133.92 (CH), 135.15 (C), 137.04 (C), 137.62 (C), 152.99 (C), 153.38 (C), 198.55 (C). **^{1}H-NMR (500 MHz, DMSO-d_6): 5b**, δ (ppm) = 0.82 (t, 3H, CH_3), 1.12 (m, 2H, CH_3-CH_2), 1.39 (m, 2H, N-CH_2-CH_2), 3.25 (t, 3H, N-CH_2), 4.70 (s, 2H, Ar-CH_2), 7.32-7.39 (m, 1H, Ar*H*), 7.41-7.48 (m, 2H, Ar*H*), 7.50-7.61 (m, 3H, Ar*H*), 7.63-7.75 (m, 3H, Ar*H*), 10.28 (s, 1H, N*H*); **5a**, some resolved resonances: 4.74 (d, 1H, Ar-CH_2), 4.96 (d, 1H, Ar-CH_2), 6.89 (d, 1H, O*H*), 7.19 (t, 1H, Ar*H*). **LC-MS (m/z):** 352.15 $[MH]^+$. **HRMS (m/z):** *calc.*: 352.1656, *found*: 352.1701 $[MH]^+$.

Reduction of the photoenol product mixture 5a+5b into cyclic 6

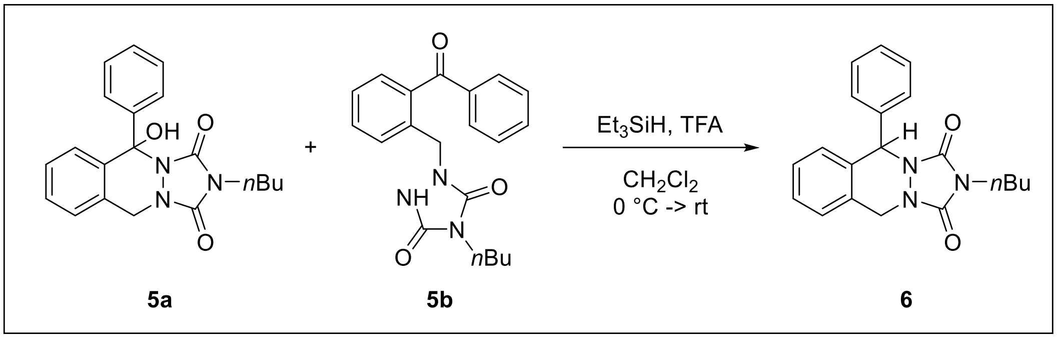

Supplementary Figure 29 | Reduction of the photoenol reaction mixture **5a**+**5b** evidencing their dynamic interrelationship.

A mixture of 4-*n*-butyl-1,2,4-triazoline-3,5-dione **1** (93.1 mg, 0.6 mmol, 1.0 eq.) and *o*-methylbenzophenone **3** (118 mg, 0.6 mmol, 1.0 eq.) in 20 mL of deoxygenated chloroform (by flushing with $N_{2\,(g)}$ for 10 min) was added to an air-tight capped headspace crimped vial placed under nitrogen atmosphere. The mixture was placed in a custom-built photoreactor and irradiated with 3 x 36 W ARIMED B6 compact fluorescent lamps (λ_{max} = 320 nm) to give a clear faint yellow solution after 2.5 h. Solvent removal *in vacuo* afforded a yellow oil containing the equilibrium mixture of **5a** and **5b**, together with traces of unreacted **3**. The obtained crude reaction mixture was next dissolved in 5 mL of anhydrous dicholoromethane and added dropwise at 0 °C to a cooled solution of triethylsilane (1.15 mL, 7.2 mmol, 6.0 eq.) and trifluoroacetic acid in 5 mL of anhydrous dichloromethane. The mixture was allowed to react for 30 minutes at 0°C, followed by overnight stirring at room temperature before being quenched with 1 M aqueous sodium hydroxide (approx. 20 mL) to pH = 7. The yellow suspension was phase-separated and the aqueous phase washed with dichloromethane (15 mL). The combined organic phases were washed with brine (30 mL), dried over magnesium sulfate and concentrated *in vacuo*. The resulting yellow oil was purified via column chromatography (silica, hexane:ethyl acetate 9:1 with a gradient to 4:1) to give unreacted **3** (14 mg – 12 %, R_F (hexane:ethyl acetate 4:1) = 0.52) together with the cyclic reduction product **6** (169 mg – 84 %, R_F (hexane:ethyl acetate 4:1) = 0.15) as a white waxy solid. **^{1}H-NMR (500 MHz, CDCl$_3$):** δ (ppm) = 0.81 (t, 3H, C*H*$_3$), 1.09 (m, 2H, C*H*$_2$-CH$_3$), 1.50 (m, 2H, N-CH$_2$-C*H*$_2$), 3.46 (m, 2H, N-C*H*$_2$-CH$_2$), 4.65 (d, 1H, Ar-C*H*$_2$), 5.06 (d, 1H, Ar-C*H*$_2$), 6.12 (s, 1H, Ar-C*H*), 7.12 (d, 1H, Ar*H*), 7.22-7.38 (m, 8H, Ar*H*). **^{13}C-NMR (500 MHz, CDCl$_3$):** δ (ppm) = 13.46 (CH$_3$), 19.41 (CH$_2$), 29.69 (CH$_2$), 38.98 (CH$_2$), 45.98 (CH$_2$), 60.03 (CH), 126.56 (CH), 127.81 (CH), 217.85 (CH), 128.45 (CH), 128.54 (CH), 128.57 (CH), 128.65 (C), 128.75 (CH), 132.75 (C), 138.32 (C), 153.62 (C), 154.62 (C). **LC-MS (m/z):** 336.10 [MH]$^+$. **HRMS (m/z):** *calc.*: 336.1707, *found*: 336.1708 [MH]$^+$.

Alder-ene addition of 1 to 7 in the dark

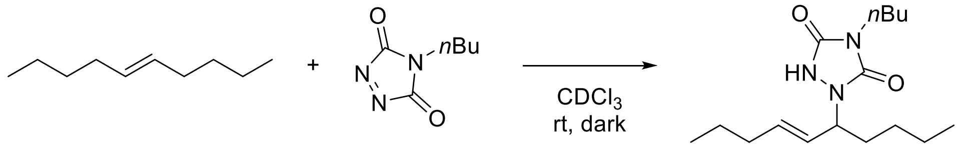

Supplementary Figure 30 | Thermal Alder-ene addition reaction of **1** to **7**.

A solution of 4-*n*-butyl-1,2,4-triazoline-3,5-dione **1** (9.31 mg, 0.06 mmol, 1.0 eq.) in 0.2 mL deuterated chloroform was added to *trans*-5-decene **7** (8.42 mg, 0.06 mmol) in 0.2 mL deuterated chloroform and stirred in the dark at room temperature. Complete discoloration was observed after 20 minutes to give a colorless solution containing the addition product **8** in quantitative yield. **^{1}H-NMR (400 MHz, $CDCl_3$):** δ (ppm) = 0.84-0.97 (m, 9H, 3 x CH_3), 1.20-1.46 (m, 8H, 3 x CH_3-CH_2 + N-CH-CH_2-CH_2), 1.58-1.77 (m, 4H, N-CH-CH_2 + N-CH_2-CH_2), 2.01 (q, 2H, CH=CH-CH_2), 3.53 (t, 2H, N-CH_2), 4.50 (q, 1H, N-C*H*), 5.41 (m, 1H, C*H*=CH-CH), 5.73 (m, 1H, CH=C*H*-CH), 8.53 (s, 1H, N*H*).

Light-controlled reaction manifold

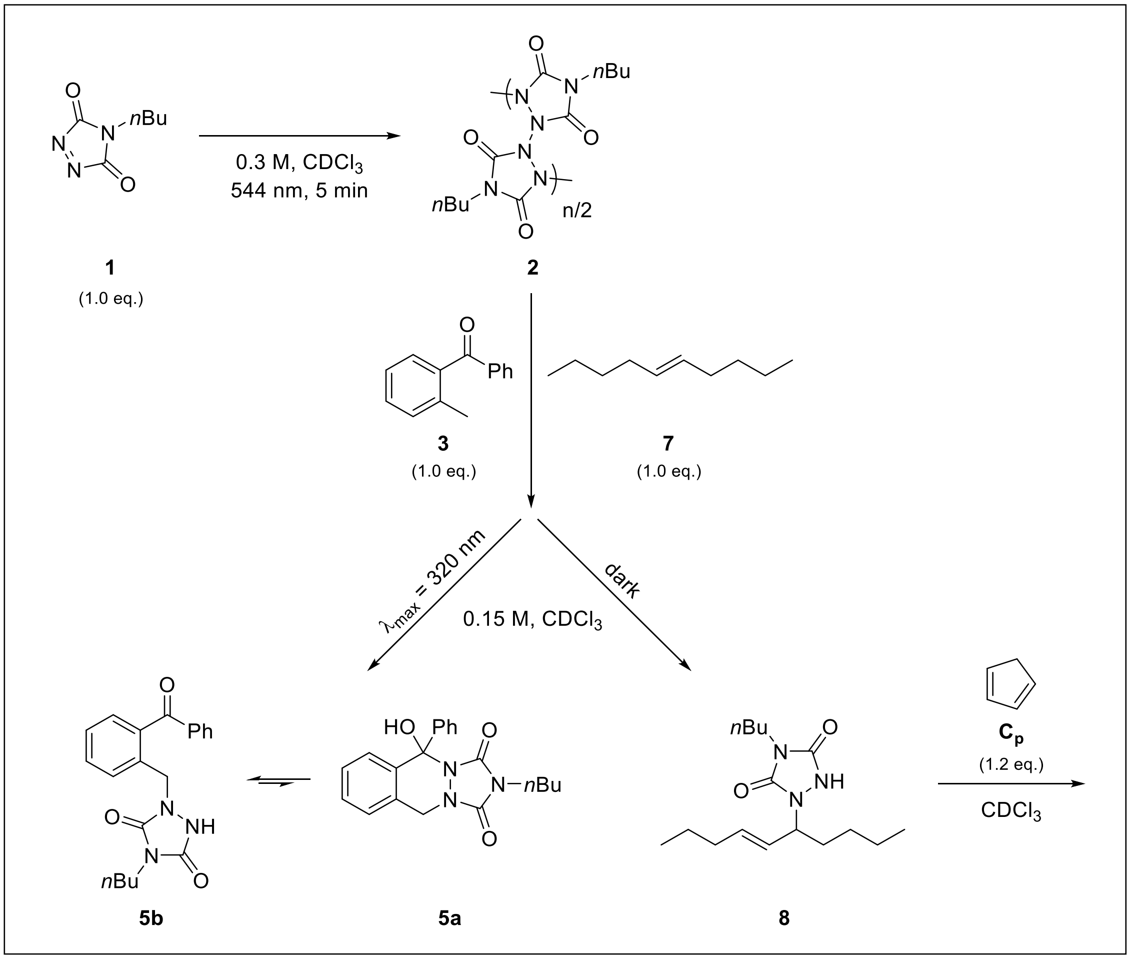

Supplementary Figure 31 | Schematic representation of the designed light-controlled reaction manifold. Visible light-irradiation of **1** enables a UV-light-switchable reaction selectivity between photoenol products **5a**+**5b** and thermal addition product **8**.

Preparation of a 0.30 M stock solution of ***1***

An air-tight crimped headspace vial with septum containing **1** (186.2 mg, 1.20 mmol, 1.0 eq.) was placed under $N_{2\,(g)}$ atmosphere. To this, 4.0 mL of deoxygenated (by flushing with $N_{2\,(g)}$ for 20 min) deuterated chloroform was added.

Preparation of a 0.30 M stock solution of ***3*** *and* ***7***

An air-tight crimped headspace vial with septum containing a mixture of *o*-methylbenzophenone **3** (235.5 mg, 1.20 mmol, 1.0 eq.) and *trans*-5-decene **7** (168.3 mg, 1.20 mmol, 1.0 eq.) was placed under $N_{2\,(g)}$ atmosphere. To this, 4.0 mL of deoxygenated (by flushing with $N_{2\,(g)}$ for 20 min) deuterated chloroform was added.

Preparation of a 0.36 M stock solution of ***C_p***

An air-tight crimped headspace vial with septum containing cyclopentadiene **C_p** (95.2 mg, 1.44 mmol, 1.2 eq.) was placed under $N_{2\,(g)}$ atmosphere. To this, 4.0 mL of deoxygenated (by flushing with $N_{2\,(g)}$ for 20 min) deuterated chloroform was added.

Procedure

The stock solution of **1** was divided into portions of 0.2 mL over several crimp neck vials (0.7 mL), quickly flushed with $N_{2\,(g)}$ and crimped air-tight. The solutions of **1** were next placed in the wavelength-tunable laser sample holder and irradiated at 544 nm (4.0 mW cm^{-2}, 100 Hz) for 5 minutes to give a clear colorless solution of **2**. Whilst the visible laser light was kept switched on, 0.2 mL of the stock solution containing **3** and **7** was added through the septum of the crimp neck vial. The resulting mixture was then immediately transferred into a custom-built photoreactor (25 °C) and irradiated at λ_{max} = 320 nm for 2 h (3 x 36 W ARIMED B6 compact fluorescence lamps) to initiate the formation of photoproduct **5a**+**5b** (***A***, Supplementary Figure 32). After UV-light irradiation, the mixture was kept in the dark (18 °C) for 8 h thereby enabling the thermal TAD-reaction to proceed to give TAD-adduct **8** (***B***). Next, the sample was again placed into the wavelength-tunable laser sample holder and irradiated at 544 nm (4.0 mW cm^{-2}, 100 Hz) for 1 h to give a non-reactive mixture without the formation of any products (***C***). To demonstrate the possibility to re-initiate the on/off switchability of the reaction manifold, the sample was again transferred into the photoreactor and subjected to UV-light for 4 h (***D***), followed by an additional 8 h of standing in the dark (***E***).

At each point (i.e. ***A***, … , ***E***), 0.2 mL of the **C_p** stock solution was injected into the samples in order to lock-in all TAD-concentrations upon standing in the dark. In other words, all *in situ* regenerated TAD is now kinetically trapped into the **Cp-1** Diels-Alder adduct without affecting the concentrations of the formed reaction products. As a result, the reaction outcome at each point during the experiment can be determined via offline ^{1}H-NMR analysis by integration of well resolved signals (cfr. Supplementary Figure 17). Furthermore, each experiment (i.e. ***A***, … , ***E***) was reproduced in threefold and the integrations were averaged out (refer to Supplementary Figure 32).

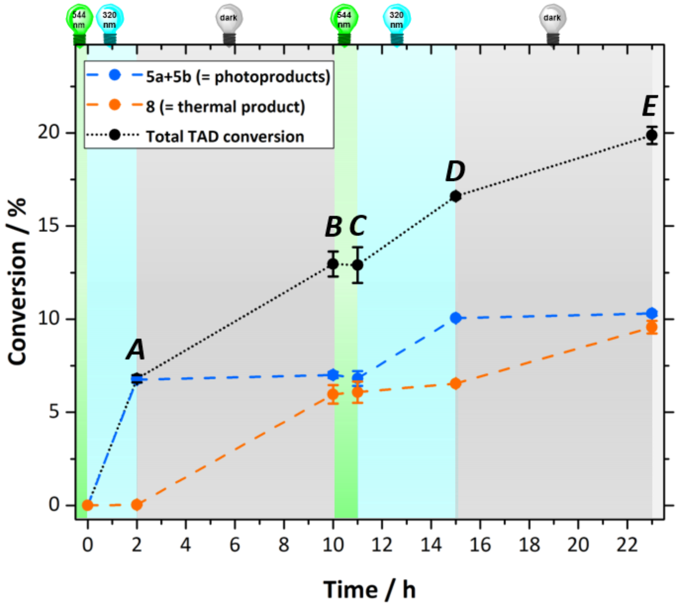

Supplementary Figure 32 | Switchable selectivity of the light-driven reaction manifold (containing an equimolar mixture of **2**, **3** and **7**) upon irradiation with UV-light (2 h, point ***A***) followed by standing in the dark (8 h, point ***B***). No reaction is observed during a one-hour period of visible light irradiation (point ***C***) whilst a second on/off cycle demonstrates the possibility to re-activate the system (point ***D*** and ***E***). The yield of the photoproducts **5a+5b**, yield of thermal adduct **8** and the overall TAD conversion were determined via ^{1}H-NMR. Traces (i.e. 2 %) of a 1:2 adduct of **1** with **3** were also detected.

Supplementary References

1. Fast DE, Lauer A, Menzel JP, Kelterer A-M, Gescheidt G, Barner-Kowollik C. Wavelength-Dependent Photochemistry of Oxime Ester Photoinitiators. *Macromolecules* **50**, 1815-1823 (2017).

2. Tuten BT, Menzel JP, Pahnke K, Blinco JP, Barner-Kowollik C. Pyreneacyl sulfides as a visible light-induced versatile ligation platform. *Chem. Commun.* **53**, 4501-4504 (2017).

3. Billiet S*, et al.* Triazolinediones enable ultrafast and reversible click chemistry for the design of dynamic polymer systems. *Nat. Chem.* **6**, 815-821 (2014).
